# Effects of conjugated linoleic acid supplementation on serum levels of interleukin-6 and sirtuin 1 in COPD patients

**Published:** 2020

**Authors:** Mohammad Reza Aslani, Somaieh Matin, Ali Nemati, Mehran Mesgari-Abbasi, Saeid Ghorbani, Hassan Ghobadi

**Affiliations:** 1 *Lung Inflammatory Diseases Research Center, Faculty of Medicine, Ardabil University of Medical Sciences, Ardabil, Iran*; 2 *Neurogenic Inflammation Research Center, Mashhad University of Medical Sciences, Mashhad, Iran*; 3 *Internal Medicine Department, Faculty of Medicine, Ardabil University of Medical Sciences, Ardabil, Iran*; 4 *Biochemistry and Nutrition Department, Faculty of Medicine, Ardabil University of Medical Sciences, Ardabil, Iran*; 5 *Drug Applied Research Center, Tabriz University of Medical Sciences, Tabriz, Iran*; 6 *Medical student, Faculty of Medicine, Ardabil University of Medical Sciences, Ardabil, Iran*; 7 *Internal Medicine Department (Pulmonary Division), Faculty of Medicine, Ardabil University of Medical Sciences, Ardabil, Iran*

**Keywords:** Conjugated linoleic acid, COPD, Sirtuin 1, IL-6, BODE index

## Abstract

**Objective:**

Chronic obstructive pulmonary disease (COPD) is characterized by systemic inflammation and accelerated inflammaging of the lungs. Some studies showed that conjugated linoleic acid (CLA) has anti-inflammatory effects. The aim of the present study was to evaluate the effect of CLA supplementation on serum levels of interleukin (IL)-6 and sirtuin1 (SIRT1) in patients with COPD.

**Materials and Methods:**

82 patients with stable COPD were enrolled in a double blind clinical trial. Subjects were randomly assigned to two groups: placebo (n=42) and 3.2 g CLA daily supplementation (n=40). Forced expiratory volume in one second (FEV1%), BODE index, and serum levels of IL-6, and SIRT1 were measured at the baseline and six weeks after the intervention. In addition, the study parameters in the two groups were compared based on the Global Initiative for Chronic Obstructive Lung Disease (GOLD) criteria.

**Results:**

After supplementation with CLA, serum levels of IL-6 and BODE index significantly decreased (p<0.05 and p<0.001, respectively). In addition, serum levels of SIRT1 (p<0.01) and FEV1 (p<0.001) significantly increased in the supplementation group. Based on GOLD criteria, the increase in SIRT1 and the decrease in IL-6 serum levels were found to be statistically significant in stages III and IV in the supplementation group (p<0.05 and p<0.01, respectively)

**Conclusion:**

Supplementation with CLA can modify the inflammatory markers and improve the health status of COPD patients. The results suggest that CLA supplementation in COPD patients can be useful in the management of the disease.

## Introduction

Chronic obstructive pulmonary disease (COPD) is the third leading cause of death in the world, resulting in more than 3 million deaths by 2020 (Andersen et al., 2011 ▶). It is estimated that in the next 10 years, COPD deaths will increase by 30% if there is no preventive action (Chun, 2015 ▶). The main feature of COPD is the airflow limitation, which is usually progressive and is associated with the chronic inflammatory response of the airways to gases and noxious particles (McDonough et al., 2011 ▶). Important factors in the pathogenesis of COPD are airway inflammation, protease-antiprotease imbalance and oxidative stress (Fischer et al., 2011 ▶). COPD not only primarily affects the lungs, but is also associated with a low-grade chronic systemic inflammation (Amani et al., 2017 ▶). In the peripheral blood of patients with COPD, elevated levels inflammatory mediators such as interleukin (IL)-6, IL-1β, tumor necrosis factor-alpha (TNF-α), and C-reactive protein (CRP) were shown (Gan et al., 2004 ▶; Ghobadi et al., 2017 ▶). IL-6 is a strong stimulus for production of CRP and plays a key role in the pathophysiology of malnutrition of COPD patients (Ferrari et al., 2013 ▶). Also, in the development of systemic inflammation in COPD, various factors, including adipocytes, are involved (Zhang et al., 2016 ▶). In the pulmonary inflammatory process, it was reported that adipocytokines have a key role in airway inflammation (Aslani et al., 2016a ▶; Aslani et al., 2016b ▶). Some studies showed that various adipocytokines such as leptin, adiponectin, and visfatin are candidate biomarkers for lung inflammatory diseases (Aslani et al., 2017 ▶; Keyhanmanesh et al., 2018 ▶).

The progress of symptom in COPD patients is slow and is generally seen in elder population (Ito and Barnes, 2009). Accordingly, considerable evidence emphasizes accelerated aging in the lungs of COPD patients (Ito and Barnes, 2009 ▶; Yanagisawa et al., 2017 ▶). Many aging characteristics are evident in patients with COPD including mitochondrial dysfunction, cellular senescence, stem cell lost, impaired autophagy, and a low-grade chronic inflammation (Carollo et al., 2018 ▶). Under oxidative stress conditions, accelerated aging may result in a defective performance of some endogenous antiaging molecules such as sirtuins (Barnes, 2016 ▶). Sirtuins are a family of highly conserved protein deacetylases and their activity depends on nicotinamide adenine dinucleotide (Carollo et al., 2018 ▶). Sirtuin 1 (SIRT1) is a one of the most important sirtuins that protects cells from cellular damage caused by oxidative stress, and increases health and life duration (Carollo et al., 2018 ▶). Growing evidence suggests that SIRT1 levels are reduced in COPD patients, and they have a positive correlation with the severity of disease (Yanagisawa et al., 2017 ▶). 

Development of an effective treatment for patients with COPD remains a challenge. The use of other therapies, especially dietary supplementation, is a clinical requirement for patients with COPD. Conjugated linoleic acids (CLA) is lipid derived from fatty acid of ruminant animals and it attracted attentions for its effects on obesity, insulin sensitivity and body composition (Chin et al., 1992 ▶). It was identified that feeding of animals with plants such as soybean and sunflower increases the level of CLA in their milk fat. There are many biological effects for CLA including effects on atherosclerosis, carcinogenesis and immune modulation (Kelley et al., 2007 ▶; O'Shea et al., 2004 ▶). In a randomized placebo-controlled trial in subjects with COPD, we previously showed that CLA supplementation reduced serum levels of IL-1β (Ghobadi et al., 2016 ▶). 

Although there are many studies about cellular mechanisms underlying the effects of CLA supplementation on pro-inflammatory and oxidative stress markers, few clinical trials were conducted to show its effects on inflammatory markers in patients with COPD. Accordingly, the present study was designed to investigate the preventive effect of six-week treatment of CLA supplementation on the modulation of the serum concentrations of IL-6 and SIRT1, exercise tolerance and pulmonary function test (PFT) in patients with COPD. 

## Materials and Methods

**Figure 1:**
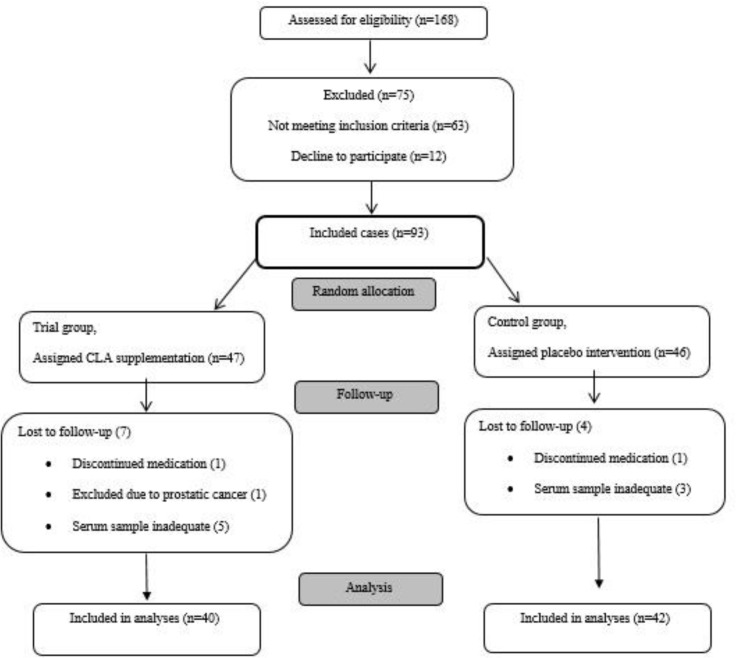
The effect of CLA-supplementation on the serum level of IL-6 and sirtuin-1 in COPD patients

In the present double-blind clinical trial study, 93 patients with stable COPD were recruited from April 2015 to December 2015. All patients were male and COPD was diagnosed according to the American Thoracic Society (ATS) guidelines (Vestbo et al., 2013 ▶). The inclusion and exclusion criteria were fully addressed previously (Amani et al., 2017 ▶). Briefly, under standard conditions, pulmonary function testing was performed using spirometry (Chest Inc., 801, Tokyo, Japan) based on ATS guidelines. Pulmonary function and biochemical tests were conducted on the same day for COPD and control subjects. Eleven patients were excluded from study analysis due to exclusion criteria. Eventually, 82 patients completed the study (42 patients in the placebo and 40 patients in the supplementation groups) (Figure 1).

The GOLD guidelines were used to categorize the severity of COPD and the six-minute walk distance (6MWD) was performed according to the guidelines of the ATS (Laboratories, 2002 ▶). Finally, we used the model described by Celli et al. to calculate BODE index (Celli et al., 2004 ▶). 

This study is the final process of a clinical trial that was previously reported the effect of CLA on IL-1β and MMP (Ghobadi et al., 2016 ▶). In the intervention group, patients received a soft gel capsule containing 3.2 g of CLA. Capsules contain c9-t11 and c12-t10 isomers made by Nortex Company (Nutrex Reaserch Inc., Oviedo, FL, USA) under the brand name Nortex. The intervention duration was 6 weeks and the control group received the same amount of placebo. To create double-blind conditions, at the beginning of the study, containers containing placebo and intervention capsules were coded with the letters A and B and the interviewers and patients were not aware of the contents of the containers.

The study was approved by Ardabil University of Medical Sciences Ethics Committee (IR.ARUMS.REC.1396.79), and all of the study participants signed written consent forms and the trial was registered in Iranian Registry of Clinical Trial “IRCT2015080823559N1”.

**Biochemical measurements**

Approximately 3-5 ml of blood samples were taken from all patients to determine serum levels of SIRT1 and IL-6 before and after intervention. Serum IL-6 (BE53061; IBL International, Hamburg, Germany) and SIRT1 (Hangzhou Eastbiopharm Co. Ltd., catalog: E20180511) levels were measured using a commercial kit. The results were reported as pg/ml.

**Statistical analysis**

Continuous variables were evaluated for normal distribution by the Shapiro-Wilk test. The results are given as the mean±SD (or median and 25th–75th percentiles). Data with normal distribution in the two study groups were compared using the t-test or the Wilcoxon‐Mann‐Whitney test for data with a skewed distribution. In order to calculate the percentage change of variables during the study period, the following was performed: (Value after treatment – value before treatment) / value before treatment X 100. A value of p<0.05 was considered significant.

## Results

Table 1 shows baseline variables for the two groups. There was no statistically significant difference in terms of age, pack/year, BMI, FEV_1_, FVC, FEV_1_/FVC, BODE, 6MWD, IL-6, and SIRT1 at the onset of the study between the two groups (p>0.05) (Table 1).

**Table 1:** Baseline parameters in each group

**Variables**	**Placebo** (n=42)	**CLA** (n=40)	***P*** **-Value**
	**Baseline**	**Baseline**	
**Age** **Pack/year** **BMI** **FEV1 (%)** **FVC (%)** **FEV1/FVC ratio** **6MWT** **mMRC** **BODE index** **IL-6 (pg/ml)** **SIRT1 (pg/ml)**	61.55±10.81 45.71±26.11 24.84±3.06 40.27±15.09 56.69±17.77 57.13±8.66 335.79±190.89 2.36±0.54 4.05±1.92 4.05 (3.14-5.24) 15.30 (12-17.60)	63.82±10.58 43.40±25.11 25.46±3.85 46.14±17.80 65.93±18.84 56.29±9.86 397.92±141.50 2.58±0.54 4.22±1.88 3.83 (2.60-4.82) 11.75 (6.30-16.90)	0.338 0.684 0.426 0.111 0.049 0.684 0.099 0.084 0.675 0.489 0.100

BMI: body mass index, FEV1: Forced expiratory volume in 1 second, FVC: Forced vital capacity, BODE: (BMI, Airway obstruction, Dyspnea, Exercise tolerance)-index, 6MWT:  *6-minute walk distance*, mMRC: Modified Medical Research Council, BODE: (BMI, Airway obstruction, Dyspnea, Exercise tolerance) index, IL-6: interleukin-6, SIRT1: sirtuin 1.

**Effects of CLA supplementation on pulmonary function test, and IL-6 and SIRT1 serum levels**

**Figure 2:**
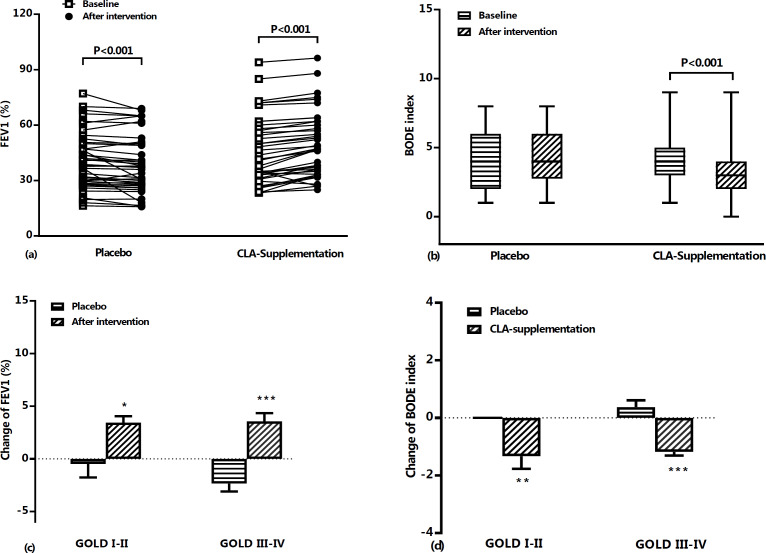
FEV1% predict (a) and BODE index (b) at baseline and after treatment period in placebo and CLA supplementation groups. Data are shown as individual value (for FEV1%) and mean±SD (or median and 25th–75th percentiles) values of percent changes in FEV1% predict (c) and BODE index (d) based on GOLD criteria in placebo and CLA supplementation groups during the study period. FEV1; forced expiratory volume in 1 second, BODE index; (Body mass index, airflow Obstruction, Dyspnea and Exercise capacity).*: p<0.05, **: p<0.01, and ***: p<0.001

**Figure 3:**
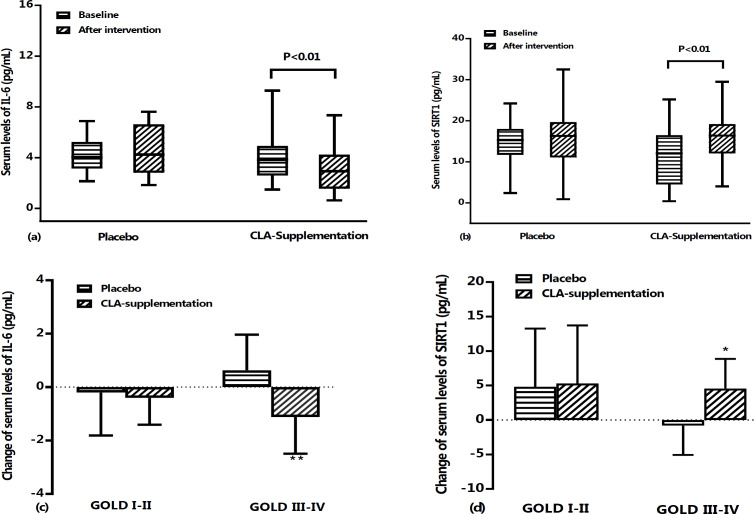
Serum levels of IL-6 (a) and SIRT1 (b) at baseline and after treatment period in placebo and CLA supplementation groups. Data are shown as mean±SD (or median and 25th–75th percentiles) values of percent changes in serum IL-6 (c) and SIRT1 (d) based on GOLD criteria in placebo and CLA supplementation groups during the study period. IL-6; interleukin 6, and SIRT1: sirtuin-1. *: p<0.05, **: p<0.01, and ***: p<0.001

No statistically significant differences were observed in serum levels of IL-6 (Figure 3a) and SIRT1 (Figure 3b) in the placebo group when baseline and after intervention data were compared. However, pulmonary function test results showed that FEV1% predict (p<0.001) (Figure 2a) significantly decreased during the study period in the placebo group. On the other hand, compared to baseline values, a significantly increased pulmonary function test of FEV1% predict (46.14±17.80 vs. 49.65±17.44, p<0.001) (Figure 2a) was found after treatment with CLA in supplementation group. BODE index results showed a significant decrease in the CLA group compared to placebo group (p<0.001, Figure 2b). Also, intervention with CLA was found to significantly decrease IL-6 (4.43±2.77 vs. 3.40±2.32, p<0.01) and increase SIRT1 levels (13.84±10.59 vs. 18.60±8.79, p<0.01). 

In the supplementation group, compared to the placebo group, there was a statistically significant decrease in percentage change of IL-6 (p<0.01) and BODE score (p<0.001, Table 2). In addition, the percentage change of FEV1 (p<0.001), FEV1/FVC ratio (p<0.001), 6MWD (p<0.001), and SIRT1 (p<0.05) were significantly higher than those of the placebo group (Table 2).

Based on the GOLD criteria, it was found that in the CLA group, value of changes in FEV1 were increased both in GOLD I and II (3.41±2.57% vs -0.48±4.07%, p<0.05) and GOLD III and IV phases (3.55±3.72% vs -2.34±4.32%, p<0.001) when compared with placebo group (Figure 2c). Also, BODE index change values between CLA and placebo groups showed a decrease in both GOLD I-II (-1.33±1.84% vs 0.00±0.00%, p<0.01) and GOLD III-IV phases (-1.18±0.58% vs 0.37±1.33%, p<0.001) (Figure 2d). However, the comparison of IL-6 and SIRT1 levels after intervention with CLA showed a significant decrease in IL-6 (p<0.01, Figure 3c) and an increase in SIRT1 (p<0.05, Figure 3d) only in stages III-IV of GOLD criteria. 

**Table 2:** Percent change in different parameters after treatment period relative to baseline values

**variables**	**Placebo (n=42)**	**CLA (n=40)**	**P value**
**FEV1 (%)**	-1.89±4.29 (-4.92%)	3.50±3.22 (9.30%)	0.000
**FVC (%)**	-0.009±5.86 (1.24%)	-2.01±10.74 (-0.33%)	0.000
**FEV1/FVC ratio**	-0.86±5.10 (-1.27%)	5.06±7.28 (10.26%)	0.000
**6MWD**	-4.97±41.29 (0.65%)	33.82±31.24 (15.76%)	0.000
**BODE index**	0.28±1.17 (11.72%)	-1.25±1.29 (-29.55%)	0.000
**IL-6 (pg/mL)**	0.35±2.20 (15.05%)	-1.03±2.40 (-11.97%)	0.004
**SIRT1 (pg/mL)**	0.53±11.92 (5.95%)	4.76±9.58 (21.50%)	0.025

FEV1: Forced expiratory volume in 1 second, FVC: Forced vital capacity, 6MWT:  *6-minute walk distance*, BODE: (BMI, Airway obstruction, Dyspnea, Exercise tolerance) index, IL-6: interleukin-6, and SIRT1: sirtuin 1.

## Discussion

The effect of CLA supplementation (3.2 g/day) in a six-week study period, was tested on pulmonary function test, GOLD stage, IL-6, and SIRT1 in patients with COPD. 

The results of the present clinical trial indicated that CLA supplementation significantly reduced serum level of IL-6 and BODE index. In addition, CLA led to elevated serum levels of SIRT1, PFT and 6MWD compared to baseline values and the placebo group. However, none of these effects were seen in the placebo group. 

Our results showed that CLA supplementation had clinical effects on patients with COPD by improving FEV1 and FEV1/FVC. In fact, in the supplementation group, the percentage of improvement in pulmonary function test was significantly higher than those of the baseline and the placebo group. Therefore, it can be concluded from the results of the study that CLA supplementation has preventive therapeutic effects in COPD patients. Interestingly, according to the results of the GOLD stage, it was found that the effects of CLA on increment of FEV1 value were significant at stages III and IV. From the results of MacRedmond et al. study, it is evident that CLA supplementation increases PFT in asthmatic patients (MacRedmond et al., 2010 ▶). Evidence suggests that CLA is able to produce clinically significant reductions in hyper-reactivity and airway inflammation by affecting adipocytokines, growth factors, inflammatory mediators, immunoglobulin, and lipid mediators (MacRedmond et al., 2010 ▶). 

High serum levels of IL-6, IL-8, IL-1β and TNF-α were seen in patients with COPD, which actually represents systemic inflammation (Wouters et al., 2007 ▶). In the pathogenesis of COPD, IL-6 and IL-8 play a key role in stable and exacerbation condition (Knobloch et al., 2010 ▶). Also, in some human studies, it was shown that increased IL-6 is associated with pulmonary impairment in patients with COPD (Wei et al., 2015 ▶). By affecting liver cells, IL-6 can act as a crucial cytokine in the production of a variety of acute phase proteins such as CRP and fibrinogen (Wei et al., 2015 ▶). It was shown that even stable COPD patients have high levels of CRP, leukocytes, fibrinogen and platelets (Sin and Man, 2008 ▶). IL-6 plays an active role by attracting neutrophils to the airways, as well as regulating several pathways involved in the progression of inflammation in the airways (Ferrari et al., 2013 ▶). Although the precise mechanism of IL-6 action in lung tissue destruction is not clear, it is imagined that IL-6 by tissue deposition, and producing protease and antibody complexes, causes destruction of lung tissue (Ferrari et al., 2013 ▶). 

In this study, CLA supplementation resulted in a decrease in IL-6 serum levels, while no significant changes were observed in the placebo group. We also found that CLA supplementation results in a significant increase in 6MWD and decreased the BODE score in the CLA supplementation group when compared with baseline value as well as the placebo group. It has been identified that there was an association between increase IL-6 and reduction of physical performance of COPD patients, although the causal relation is unclear (Ferrari et al., 2013 ▶). Brinkely et al. demonstrated that high levels of IL-6 were associated with decreased physical activity in older adults with multiple comorbidities, such as COPD independent of age, race, sex, and body composition (Brinkley et al., 2009 ▶). Also, studies conducted by Garrod et al. and Yende et al. identified that there was a negative association between IL-6 levels and 6MWD in COPD patients (Garrod et al., 2007 ▶; Yende et al., 2006 ▶). In addition, Pinto-Plata et al. showed that high levels of inflammatory markers were associated with a degree of airflow limitation, health status and functional capacity (Pinto-Plata et al., 2012 ▶). 

Based on our study, improvement in pulmonary function test and BODE score in the supplementation group was probably due to a reduction in IL-6 levels. Research on food immunology in animal study has revealed an important role for the CLA regimen in reducing inflammation (Changhua et al., 2005 ▶). CLA supplementation was shown to be able to reduce levels of IL-6, IL-1β, and TNF-α and increase IL-10 levels (Changhua et al., 2005 ▶). The inhibitory effects of CLA on the production of pro-inflammatory cytokines at the molecular level are likely to be dependent on transcriptional regulation. In animal studies, it was demonstrated that the modulatory effects of CLA in the synthesis of pro-inflammatory cytokines are dependent on the PPARγ mechanism (Changhua et al., 2005 ▶). As a result of activation of PPARγ, down-regulation occurs in the signal pathways of mitogen activated protein kinase (MAPK) and nuclear factor-κB (NF-kB) (Changhua et al., 2005 ▶). CLA, possibly with its anti-inflammatory effects, was able to exert improving effects in patients with COPD. However, it should be noted that IL-6 is produced by various cells and organs in patients with COPD such as the liver, muscle, adipocytes, and lung (Sin and Man, 2008 ▶), and the effects of CLA on reducing the amount of circulating IL-6 require further studies.

We also indicated that the protein level of SIRT1 in CLA supplementation group was increased compared to baseline and placebo group, whereas in placebo group, SIRT1 level did not change during the six-week study period. Interestingly, based on GOLD criteria results, significantly elevated serum levels of SIRT1 in CLA supplementation group were only observed in stages III-IV. Yanagisawa et al. reported that protein levels of SIRT1 were significantly decreased in patients with COPD (Yanagisawa et al., 2017 ▶). It is also known that the protein levels of SIRT1 were associated with the severity of the airways obstruction and have a strong negative correlation with the amount of cigarette consumption, suggesting that oxidative stress may lead to a decrease in SIRT1 levels (Kwon and Ott, 2008 ▶). Accordingly, SIRT1 levels may be an important factor in the estimation of some disease characteristic in patients with COPD such as emphysema. On the other hand, Kim et al. showed that CLA supplementation had significant effects on the increase of SIRT1 (Kim et al., 2016 ▶). Kim et al. demonstrated that CLA supplementation in wild-type male animals led to an increase in SIRT1 values (Kim et al., 2016 ▶). In addition, in an animal study conducted by Shen and colleagues, it was also found that CLA supplementation resulted in an increase in SIRT1 activity (Shen et al., 2018 ▶), which is consistent with the current study outcomes. 

Occurrence of inflammation in the respiratory system activates various types of cells, such as macrophages, neutrophils, eosinophils, and epithelial cells that leads to the production of reactive oxygen species (ROS) (Chun, 2015 ▶). Under conditions of high ROS production, cellular ROS-induced damage occur as a result of redox imbalance (Yadav and Ramana, 2013 ▶). Various transcription factors such as activator protein-1 (AP-1) and NF-kB are activated as a result of redox activation and increase the production of pro-inflammatory molecules. SIRT1 plays an important role in modulation of redox processes (Yadav and Ramana, 2013 ▶). It was shown that SIRT1 induces pulmonary protection against cellular inflammation in response to oxidative stress by regulating various cellular processes such as autophagy, apoptosis, and mitochondrial biogenesis (Yao and Rahman, 2011 ▶). Although the precise mechanism of SIRT1 activity in patients with COPD is unclear, the increase in SIRT1 level as well as the improvement of pulmonary function and 6MWD modification as a result of CLA intervention, may be related to these changes. Our results also showed a negative correlation between the SIRT1 and IL-6 levels in the CLA supplementation group, markedly in stages III-IV of GOLD criteria. It may be deduced that in patients with COPD under intervention with CLA, various mechanisms can be activated, directly or indirectly, to improve pulmonary function and reduce inflammatory markers, which requires more investigation. 

This study demonstrated that CLA supplementation has anti-inflammatory effects mediated through suppression of IL-6 production. CLA can modulate SIRT1 levels in COPD patients. Supplementation with CLA can modify the inflammatory markers and improve the health status of COPD patients. Evaluation of other inflammatory pathways involved in patients with COPD under CLA supplementation needs further studies to determine its efficacy.
